# Ovarian cancer proteomic landscape changes across different pathological stages and biomarker-sets identified with integrative multiomics analysis

**DOI:** 10.3389/fendo.2026.1898210

**Published:** 2026-09-07

**Authors:** Yan Wang, Zheng Fang, Nuo Xu, Liang Chen, Xianquan Zhan

**Affiliations:** 1 Department of Gynecological Oncology, Shandong Cancer Hospital and Institute, Shandong First Medical University & Shandong Academy of Medical Sciences, Jinan, China; 2 Shandong Provincial Key Laboratory of Precision Oncology, Shandong Cancer Hospital and Institute, Shandong First Medical University & Shandong Academy of Medical Sciences, Jinan, Shandong, China; 3 Department of Gynecology, People’s Hospital of Gaotang County, Liaocheng, Shandong, China; 4 Shandong Provincial Key Medical and Health Laboratory of Ovarian Cancer Multiomics, School of Pharmaceutical Sciences, Shandong First Medical University, Jinan, Shandong, China; 5 Jinan Key Laboratory of Cancer Multiomics, School of Pharmaceutical Sciences, Shandong First Medical University, Jinan, Shandong, China

**Keywords:** biomarkers, chromosome stability, data-independent acquisition (DIA), genomic stability, molecular staging, ovarian cancer, predictive preventive personalized medicine (PPPM/3PM), proteomic landscape

## Abstract

**Background:**

Ovarian cancer (OV) is a highly lethal gynecological malignant tumor, with a high mortality, low survival rate, and lacking effective biomarkers. The concept of predictive, preventive, and personalized medicine (PPPM) underscores the need for early warning systems and tailored interventions, creating a demand for reliable, stage-specific biomarkers.

**Methods:**

A comprehensive proteomic analysis of 60 OV tissues across pathological stages I-IV, and 17 benign ovarian tissues were analyzed with data-independent acquisition (DIA) mass spectrometry. Moreover, these proteomic findings were integrated with TCGA/GTEx data for survival analysis and key molecules, followed by experimental validation with western blot and multiplex immunohistochemistry.

**Results:**

Totally, 1,669 differentially abundant proteins (DAPs) were significantly different in each pathological stage (I, II, III, and IV) of OVs compared to controls. DAPs in the early stage (Stage I-II) were mainly enriched in biological processes related to cell proliferation, such as cell cycle process and chromosome segregation, DAPs in advanced stages (Stages III–IV) were mainly enriched in pathways including extracellular matrix (ECM) organization and cell adhesion. Trend cluster analysis of these DAPs identified six types of trend clusters across different stages of OVs (C1–C6). PPI network analysis identified 28 hub proteins with multiple centrality algorithms. Of them, hub protein SMC2 was localized at nuclear and upregulated during early stage (I/II), which was significantly upregulated at its mRNA and protein levels in OV tissues with TCGA/GTEx, western blotting, and multiplex immunofluorescence analyses, and had a significant relationship with reduced overall survival (HR = 1.31, 95% CI: 1.12–1.55, p = 0.0011) with Kaplan-Meier survival analysis.

**Conclusion:**

This study provided the large-scale stage-resolved proteomic atlas of OVs, identifies a 28-protein core module governing cell cycle and genome stability, and reveals SMC2 plays crucial roles in OVs.

## 1 Introduction

Ovarian cancer (OC) is a highly lethal gynecological malignant, malignancy, among which epithelial ovarian cancer (EOC) accounts for approximately 90% of cases, with high-grade serous ovarian carcinoma (HGSOC) being the most common subtype. Globally, there are about 290,000–320,000 new cases annually, and more than 200,000 women die from this disease. In the United States alone, an estimated 21,010 new cases and 12,450 deaths are projected for 2026. Due to the absence of specific early symptoms and the lack of effective screening strategies, over 70% of OC patients are diagnosed at an advanced stage (FIGO III–IV), with a five-year survival rate below 30% (1–3). The standard therapy for OC patients consists of cytoreductive surgery combined with platinum-based systemic chemotherapy. Optimal cytoreductive surgery is the cornerstone of treatment, with the primary goal of achieving microscopic residual-free resection (R0 resection). R0 resection is significantly associated with long-term survival, yielding a five-year survival rate of 60-70%, compared to only 20-30% for R1/R2 resection (4). Because of the unique “seed-and-soil” pattern of tumor dissemination, especially in advanced-stage patients, extensive involvement of the peritoneum, omentum, mesentery, and diaphragm poses a great challenge to achieving R0 resection (5, 6).

Although surgery and first-line platinum-based chemotherapy can achieve initial remission, most patients relapse within 6–24 months after surgery, with a cumulative three-year relapse rate as high as 85%. Patients with recurrent OV gradually develop platinum resistance, and recurrent disease is generally considered incurable, representing a major clinical challenge. For chemosensitive relapses, secondary cytoreductive surgery may benefit selected patients, whereas those with resistant disease derive little benefit from further surgery and have a dismal prognosis. Tumor heterogeneity (including both inter-tumor and intra-tumor heterogeneity) is a key driver of treatment failure and metastasis, and is particularly prominent in HGSOC, where chromosomal instability (CIN) drives copy number variations, leading to extensive subclonal heterogeneity (7, 8). Moreover, the tumor microenvironment plays an essential role in chemoresistance and disease progression (9, 10); cancer stem cells (CSCs) survive therapeutic pressure and sustain continuous tumor growth, while tumor-associated macrophages (TAMs) contribute to immunosuppressive and pro-tumorigenic signals (11).

Predictive, preventive, and personalized medicine (PPPM) has emerged as a pivotal direction in human healthcare, advocating precise control throughout the entire disease continuum – from early molecular warning and stratified management to targeted therapy (12, 13). Currently used serum biomarkers such as CA125 and HE4, while helpful for diagnosis, have limited sensitivity and specificity for early detection (sensitivity ~50-70%) and lack molecular indicators that reflect dynamic tumor progression. In current clinical practice, preoperative assessment still relies heavily on imaging (CT/MRI) and serum CA125 levels, but these tools have limited ability to predict optimal (R0) cytoreduction, and conventional scoring systems show only modest performance. Furthermore, there is a lack of reliable biomarkers to predict chemotherapy response or monitor minimal residual disease, making it difficult to timely adjust treatment intensity or switch to alternative regimens. Therefore, systematically mapping the proteomic landscape across different stages of OV and identifying key regulatory molecules is essential for achieving early diagnosis and personalized therapy.

Advances in quantitative proteomics, particularly data-independent acquisition (DIA), provide high-precision tools for biomarker discovery. Compared to conventional data-dependent acquisition (DDA), the diaPASEF mode of DIA significantly improves detection reproducibility and coverage depth for low-abundance proteins, making it especially suitable for molecular subtyping of highly heterogeneous tumors like OV (14). Previous studies have implicated aberrant activation of cell cycle regulatory pathways (e.g., DNA Areplication, chromosome segregation) as a core driver of malignant progression in OV. However, most investigations have focused on single stages or isolated molecules, lacking systematic cross-stage analysis and clinical translation. Against this backdrop, structural maintenance of chromosomes 2 (SMC2), a key cell cycle regulatory protein, has come into focus due to its central role in chromosome condensation, DNA repair, and cell cycle regulation. Malignant cells possessing CIN rapidly acquire somatic copy-number alterations (SCNAs) during proliferation, generating intratumoral genetic heterogeneity. This accelerates phenotypic adaptation under selective pressures such as therapy, ultimately contributing to poor clinical outcomes (15, 16). CIN refers to an elevated rate of chromosome mis-segregation during mitosis, generating structural and numerical chromosomal aberrations that sustain tumor evolution. Mechanistically, centrosome anomalies lead to numerical and/or structural chromosomal alterations, which in turn promote genomic instability (17). In HGSOC, this CIN-driven process generates widespread SCNAs that disrupt tumor suppressor genes and oncogenes. Quantitative analyses have demonstrated that HGSOC is the most SCNA-driven cancer across 14 solid tumor types, with SCNAs exerting pronounced effects on gene expression and signaling pathways (18). At the single-cell level, CIN drives rapid acquisition of subclonal copy-number variations (CNVs), generating substantial intratumoral heterogeneity and enabling divergent clonal trajectories that underpin therapy resistance and poor clinical outcomes (8).

This study integrated DIA quantitative proteomics data from different stage of OV tissues (FIGO I-IV, n=60) and benign controls (n=17), systematically analyzed the protein expression profiles across various cancer stages and benign tissues to identify key molecules with stage-specific expression trends, elucidate their functions in pathways such as cell cycle and genomic stability, and explore their translational potential within the PPPM framework by incorporating survival analysis and experimental validation. This study aims at discovery of novel biomarkers for early diagnosis, potentially advancing OV management toward PPPM practice in OVs.

## 2 Materials and methods

### 2.1 Sample specimen

In this study, all ovarian tissue samples were collected from Department of Ovarian Cancer of Shandong Cancer Hospital. This study was conducted in accordance with the Declaration of Helsinki, and the protocol was approved by the Institutional Review Board of Shandong Cancer Hospital (Approval No.: [SDTHEC 202503004]). Informed consent of each participant was obtained. A total of 77 ovarian tissue specimens (**Supplementary Table 1**) were included as the discovery cohort for DIA proteomic profiling. This cohort consisted of 17 benign ovarian tumors (BT) serving as the control group and 60 cases of primary HGSOC as the experimental group. All carcinoma tissues were clinically and pathologically diagnosed and staged according to the 2014 FIGO staging system for OV, including stages I (n = 6), II (n = 14), III (n = 20), and IV (n = 20). The median age of the discovery cohort was 54 years (range 12–72). Preoperative serum CA125 and HE4 levels were substantially elevated in HGSOC patients compared to benign controls, with median values of 429 U/mL vs. 18.4 U/mL for CA125 and 236 pmol/L vs. 42.9 pmol/L for HE4 (**Supplementary Table 1**). Among the 60 HGSOC patients, 12 had received neoadjuvant platinum-based chemotherapy, all of whom were in stages III–IV, and 4 had a history of breast cancer (**Supplementary Table 1**). Each benign ovarian tumor was diagnosed with rigorous histological evaluation to ensure it was solely benign lesions without any concomitant *in situ* or invasive malignant components. Tissue specimen used for proteomic analysis were immediately snap-frozen in liquid nitrogen following surgical resection and stored at -80 °C. Moreover, to experimentally validate proteomic results, independent sample subsets were selected from those 77 ovarian tissue samples for western blot (WB; frozen samples) and multiplex immunohistochemistry (mIHC; FFPE sections) analyses.

### 2.2 Sample preparation

Proteins were extracted with a lysis buffer (100 mM Tris-HCl, pH 8.5, containing 1.5% SDS). Tissue samples were homogenized with a tissue homogenizer, followed by denaturation (95 °C; 15 min), ultrasonication, and centrifugation. The supernatant containing proteins was treated with acetone precipitation. The resulting protein pellets were resuspended in solubilization buffer (8 M urea, 100 mM Tris-HCl, pH 8.5), and protein content was measured with the BCA assay (Thermo Fisher). For tryptic digestion, equal protein amounts from each sample were adjusted to the same volume with the solubilization buffer, followed by reduction with TCEP [Tris(2-carboxyethyl) phosphine] and alkylation with CAA (2-chloroacetamide) (37 °C, 1 h). Also, the urea concentration was diluted to < 2 M by adding 100 mM Tris-HCl solution, followed by protein digestion with trypsin (ratio of trypsin-to-protein mass = 1:50) (overnight, 37 °C, agitation). Then protein digestion was stopped with trifluoroacetic acid (TFA), followed by centrifugation (12,000 g), and desalted with homemade SDB StageTips, vacuum-dried, and then the desalted supernatant was stored (-20 °C).

### 2.3 LC-MS/MS

The tryptic peptide was analyzed with LC-MS/MS in Thermo UltiMate 3000 RSLCnano system coupled to a timsTOF Pro mass spectrometer (Bruker) (19). The tryptic peptide samples were loaded onto a C18 trap column (75 µm × 2 cm, 3 µm particle size, 100 Å pore size; Thermo) and separated on an analytical column (75 µm × 15 cm, 1.7 µm particle, 100 Å pore; IonOpticks). The LC analytical gradient was established with mobile phases A (0.1% formic acid in water) and B (0.1% formic acid in acetonitrile). The data acquisition mode was set as diaPASEF – DIA, capillary voltage 1500 V, MS1 and MS2 scan ranges *m/z* 100-1700, ion mobility range 0.6-1.6 Vs/cm², accumulation time 50 ms, and ramp time 50 m s. diaPASEF acquisition windows were defined with timsControl software using *m/z* value and ion mobility distributions. Collision energy was set as linear decrease from 59 eV at 1/K0 = 1.6 Vs/cm² to 20 eV at 1/K0 = 0.6 Vs/cm² (20, 21).

### 2.4 Database search with DIA data

The DIA-NN (version 1.9.2) in library-free mode was used to process DIA raw data (14, 22). The searching parameters were set as human protein database from UniProt (2024-08-07, 20,654 entries) (23), precursor ion generation for *in silico* prediction of the spectral library, enzyme trypsin/P with up to two missed cleavages permitted, fixed modification carbamidomethyl (C), variable modifications oxidation (M) and acetylation (protein N-terminal), mass tolerances 15 ppm for both MS1 and MS2, match between runs (MBR), heuristic protein inference, MaxLFQ algorithm for label-free quantification were enabled (24, 25). MaxLFQ derives protein abundance ratios from the maximum intensity values of identified peptides across samples, which improves quantification accuracy particularly for proteins with few peptide identifications. Its statistical basis assumes that peptide intensities follow a log-normal distribution, and that abundance estimates remain reliable even with missing values when multiple peptides are available (26). The false discovery rate (FDR)= 1% at the protein level, and the other parameters as default primarily used.

### 2.5 Proteomics data analysis

#### 2.5.1 Protein quantification and differential analysis

The “pg_matrix.tsv” file from the DIA-NN output was used for protein-level quantitative analysis (22). Subsequent bioinformatic analysis and visualization were conducted with R (v4.4.3). Missing value was imputed from normal distribution representing mass spectrometry detection limit for respective samples (downshift = 1.8 standard deviations, width = 0.25 standard deviations). Proteins with valid quantitative values in at least 50% of the samples within any experimental group were retained for comparative analysis. Duplicate protein entries were collapsed by retaining the entry with the highest number of unique peptides for each gene symbol. Entries identified solely through shared peptides without any unique peptides were removed from the dataset prior to downstream analysis. Each differentially abundant protein (DAP) was identified in each stage of OVs relative to controls.

#### 2.5.2 Functional characteristics analysis

To investigate the potential biological functional characteristics of DAPs in OV, functional annotation and enrichment analysis were performed using the clusterProfiler package (version 4.10.0) in R (version 4.4.3), with GO (27), KEGG pathway (28) databases. COG functional categories were obtained through the eggNOG database, while Pfam domains and subcellular localization (SL) annotations were retrieved from the UniProt database, which provides manually curated localization information based on published experimental evidence. Significantly functional terms and pathways were determined with Fisher’s exact test (P < 0.05), with the Rich factor serving as a metric to evaluate the degree of enrichment.

#### 2.5.3 Dynamic expression trend patterns with MFUZZ analysis

To delineate the dynamic expression trend patterns of key DAPs during different stages of OVs and to support early prediction, risk warning, and precise therapeutic strategies, the MFUZZ soft clustering algorithm was utilized to analyze the trend profiles of 1,669 DAPs across different stages of OVs. MFUZZ, based on a fuzzy C-means (FCM) algorithm, is suitable to identify complex, continuous, and overlapping expression patterns in biological systems. This algorithm allows proteins to belong to multiple trend clusters with varying membership degrees, better reflecting potential multifunctional roles across different biological processes or disease stages. In this study, protein expression data were first normalized with Z-score transformation before being subjected to MFUZZ clustering. The optimal cluster number was set to c = 6, resulting in six distinct clusters (C1–C6). Each cluster represents a group of proteins with similar expression trends, facilitating the identification of dynamic expression changes across BTs and different stages (I, II, III, and IV) of OVs.

#### 2.5.4 Protein-protein interaction network analysis

The STRING database (https://string-db.org) (29) was utilized to construct PPI network of DAPs for further investigation of the functional relationships of DAPs in OV. This database integrates known and predicted protein interactions, including physical associations and functional linkages. A combined score threshold > 0.4 (STRING default medium confidence) was applied to balance sensitivity and specificity; higher thresholds were avoided as they would reduce interaction recovery and potentially exclude biologically relevant connections, particularly given the modest DAP set sizes in certain comparisons. Topological analysis and visualization of the interaction network were performed using Cytoscape (version 3.9.1). Hub proteins were identified using the CytoHubba plugin (version 0.1). The following topological parameters were calculated for each node: degree centrality (number of direct connections), betweenness centrality, and closeness centrality. Four algorithms (MCC, MNC, DMNC, and Degree) implemented in CytoHubba were used to rank proteins. These algorithms capture distinct topological features: Degree reflects local connectivity, MNC identifies nodes within densely connected neighborhoods, DMNC prioritizes bottleneck nodes, and MCC integrates both local and global topological characteristics. To balance network coverage with statistical stringency and reduce false positives, the top 100 ranked proteins from each algorithm were retained for subsequent intersection analysis.

#### 2.5.5 Validation of hub molecule expression with TCGA/GTEx via GEPIA

To enhance the accuracy of biomarker screening, the gene expression levels of the identified hub proteins were analyzed with the GEPIA (gene expression profiling interactive analysis) platform (http://gepia.cancer-pku.cn/) (30). GEPIA is a web-based tool that utilizes data from The Cancer Genome Atlas (TCGA) and the Genotype-Tissue Expression (GTEx) project. The transcriptomic data were extracted from 514 ovarian serous cystadenocarcinoma samples (426 tumor vs. 88 normal samples) in the TCGA/GTEx database. Based on its central position in the PPI network and its significantly elevated expression confirmed by GEPIA, SMC2 was prioritized for further survival analysis.

#### 2.5.6 Survival analysis

Overall survival (OS), defined as the time from diagnosis to death from any cause, was assessed using the Kaplan-Meier Plotter online tool (http://kmplot.com/analysis). Patients were divided into high- and low-expression groups according to the auto-selected best cutoff. Hazard ratios (HR) with 95% confidence intervals (CI) were calculated using the log-rank test, and P-values < 0.05 were considered statistically significant. All parameters remained at their default settings except for gene symbol (SMC2), OS, histology (serous), auto-selection of the best cutoff (enabled), and the exclusive use of the JetSet best probe set.

### 2.6 Western blotting analysis

The extracted proteins from cells or tissues were mixed with 5× protein loading buffer (4:1 volume ratio), followed by denaturation (95 °C, 10 min), and storage (-20 °C). Separation and stacking gels were prepared, and protein samples along with molecular weight standards were loaded onto the gels, followed by electrophoresis at 80V (the bromophenol blue dye front was needed to reach the interface between the stacking and separation gels), and then at 120V (the bromophenol blue dye front was needed to reach the bottom of the separation gel). After electrophoresis, the separation gel and pre-activated PVDF membranes were assembled for semi-dry transfer proteins onto PVDF membranes (1.5A; 7 min), followed by blocking with 5% skim milk in TBST (2 h; room temperature), incubation with primary antibodies (SMC2 antibody 1:1000 dilution; β-actin antibody 1:5000 dilution) (4 °C; overnight), washing with TBST (5x; 5 min each time), incubation with HRP-conjugated secondary antibodies (1:10000 dilution) (2 h; room temperature), and washing with TBST (5x; 5 min each time). ECL chemiluminescent substrate was applied for several minutes before exposure to X-ray film. ImageJ software was used to quantify the intensity of each western blotting band. β-actin was used as the internal control.

### 2.7 Multiplex IHC

Each prepared tissue section was deparaffinized with xylene, and rehydrated with a series of alcohol solutions. AR6 buffer (Akoya Biosciences) was used for antigen retrieval through microwave heating, followed by incubation (10 min) with 3% hydrogen peroxide to quench endogenous peroxidase activity. The mIHC protocol involved sequential staining cycles. Each cycle included protein blocking with 1% BSA, and then incubation with HRP (horseradish peroxidase)-conjugated secondary antibodies from mouse or rabbit (Akoya Biosciences). Signal amplification was achieved through tyramine-based covalent linkage (TSA) with opal fluorophores (1:100 dilution with 1× Plus Amplification Diluent) (Akoya Biosciences). After each opal fluorophore was covalently conjugated to target epitopes, antibodies (primary; secondary) were removed with the aforementioned antigen retrieval method. The primary antibody and Opal fluorophore sequence was anti-SMC2 (30707-1-AP, 1:400, Proteintech)/Opal 620. The spectral DAPI (Akoya Biosciences) was used to counterstain each prepared tissue section, followed by mounting with anti-fade fluorescent mounting medium (ab104135, Abcam). The PANNORAMIC SCAN II imaging system (3Dhistech, Hungary) was used to visualize the multiplex imaging (200× magnification).

### 2.8 Statistical analysis

Each quantitative data was described as mean ± SD (standard deviation), with independent biological replicates ≥3 for each experiment. For the proteomic data, proteins were retained for analysis only if quantified in at least 50% of samples within any comparison group. Missing values were treated with normal distribution that simulates the detection limit (downshift = 1.8 SD, width = 0.25 SD). Each DAPs was identified with an absolute fold change (FC) ≥ 2 (|log_2_FC| ≥ 1), and a P-value < 0.05 from two-sided Student’s t test. No multiple-testing correction was applied, as this was a discovery-phase study aimed at identifying candidate proteins rather than confirmatory hypothesis testing. In experimental validation, western blot band intensities were normalized to β-actin. For mIHC, the mean fluorescence intensity within DAPI-defined nuclear regions was measured. ANOVA (one-way analysis of variance) was utilized to determine the differences in different stages of OVs compared to benign controls. Dunnett’s *post-hoc* test was applied for pairwise comparisons against the control group. The Kaplan-Meier Plotter online tool was used for survival analysis, and between-group differences were evaluated with log-rank test. Differential SMC2 mRNA expressions in tumor compared to normal tissues in the TCGA/GTEx datasets was analyzed using the GEPIA2 platform with its default parameters. A P-value < 0.05 means statistically significance for all analyses.

## 3 Results

### 3.1 Identification of OV stage-associated DAP profiles with quantitative proteomics

**Figure 1:**
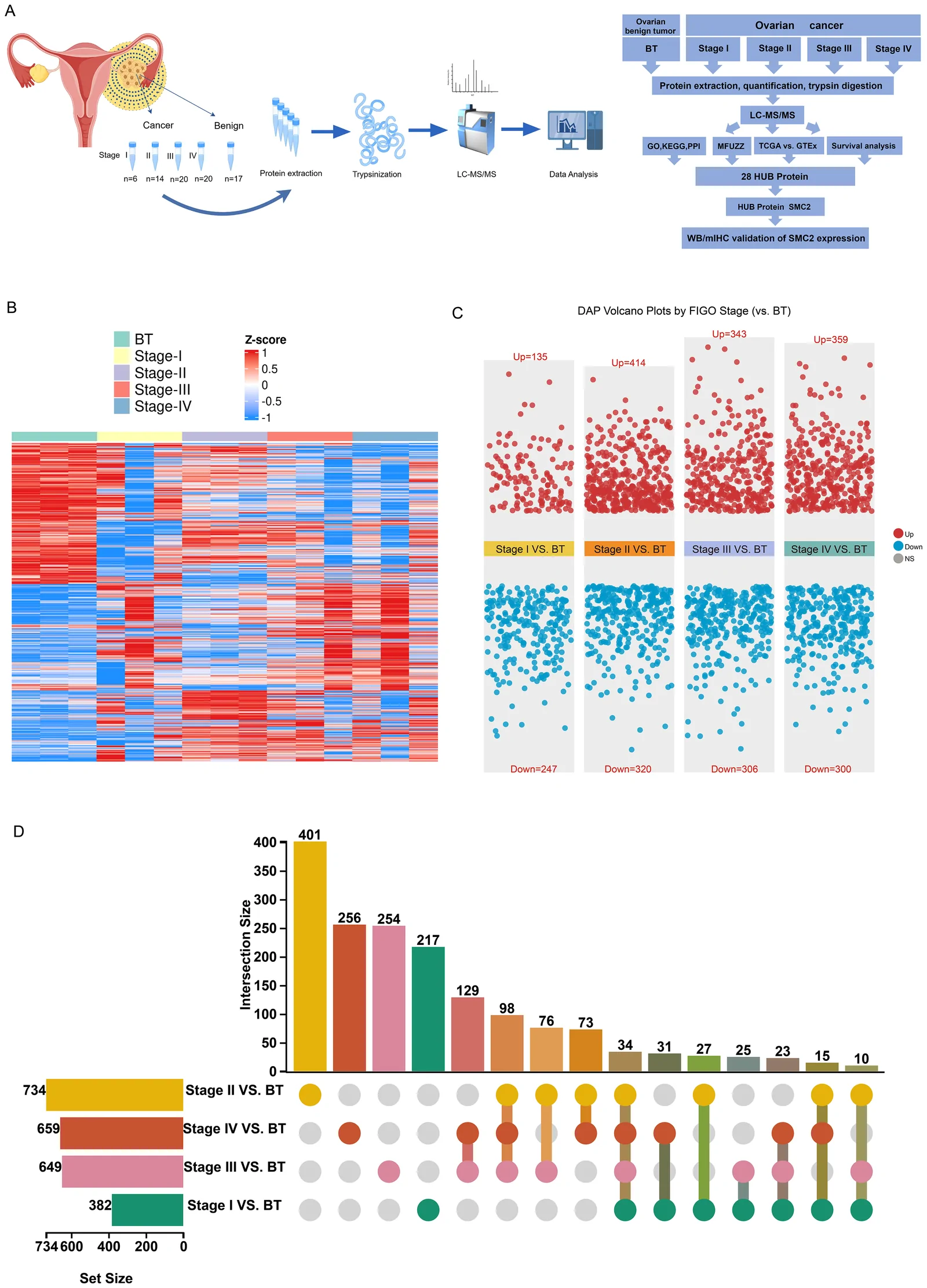
Proteomic landscape across different stages (I, II, III, and IV) of ovarian cancers. **(A)** Experimental flow chart of DIA quantitative proteomics in analysis of different stages (I, II, III, and IV) of ovarian cancers. **(B)** Unsupervised clustering heatmap of 1,669 DAPs across all samples. Color scale represents Z-score normalized protein expression. **(C)** Volcano plots of DAPs in each stage of ovarian cancers vs. BT. Each DAP was determined with thresholds (|log_2_FC| ≥ 1, P< 0.05), and its upregulation in red color and downregulation in blue color. **(D)** UpSet plot visualizing overlaps among ovarian cancer stage-specific DAPs, especially overlapped DAPs common to all stages or unique to early (I+II) or late (III+IV) stages.

DIA quantitative proteomic analysis of different stages (I, II, III, and IV) of OV tissues vs. BTs (**Figure 1A**) identified a total of 76,031 tryptic peptides derived from 8,758 proteins, including 1,669 DAPs across different stages (I, II, III, and IV) visualized with heatmap (**Figure 1B**; **Supplementary Table 2**): 382 DAPs (135 up, and 247 down) in stage I vs. BT, 734 DAPs (414 up, and 320 down) in stage II vs. BT, 649 DAPs (343 up, and 306 down) in stage III vs. BT, and 659 DAPs (359 up, and 300 down) in stage IV vs. BT. (**Figure 1C**). UpSet analysis revealed unique and overlapping DAPs in different stages: 217 proteins were exclusively altered in Stage I, 401 in Stage II, 254 in Stage III, and 256 in Stage IV (**Figure 1D**; **Supplementary Table 2**). Moreover, 27 proteins were specific to early stages (I+II), 129 to late stages (III+IV), and 34 proteins were consistently differentially expressed across all four cancer stages (**Figure 1D**; **Supplementary Table 2**).

### 3.2 Molecular functions and altered signaling pathways of stage-specific DAPs in OV

**Figure 2:**
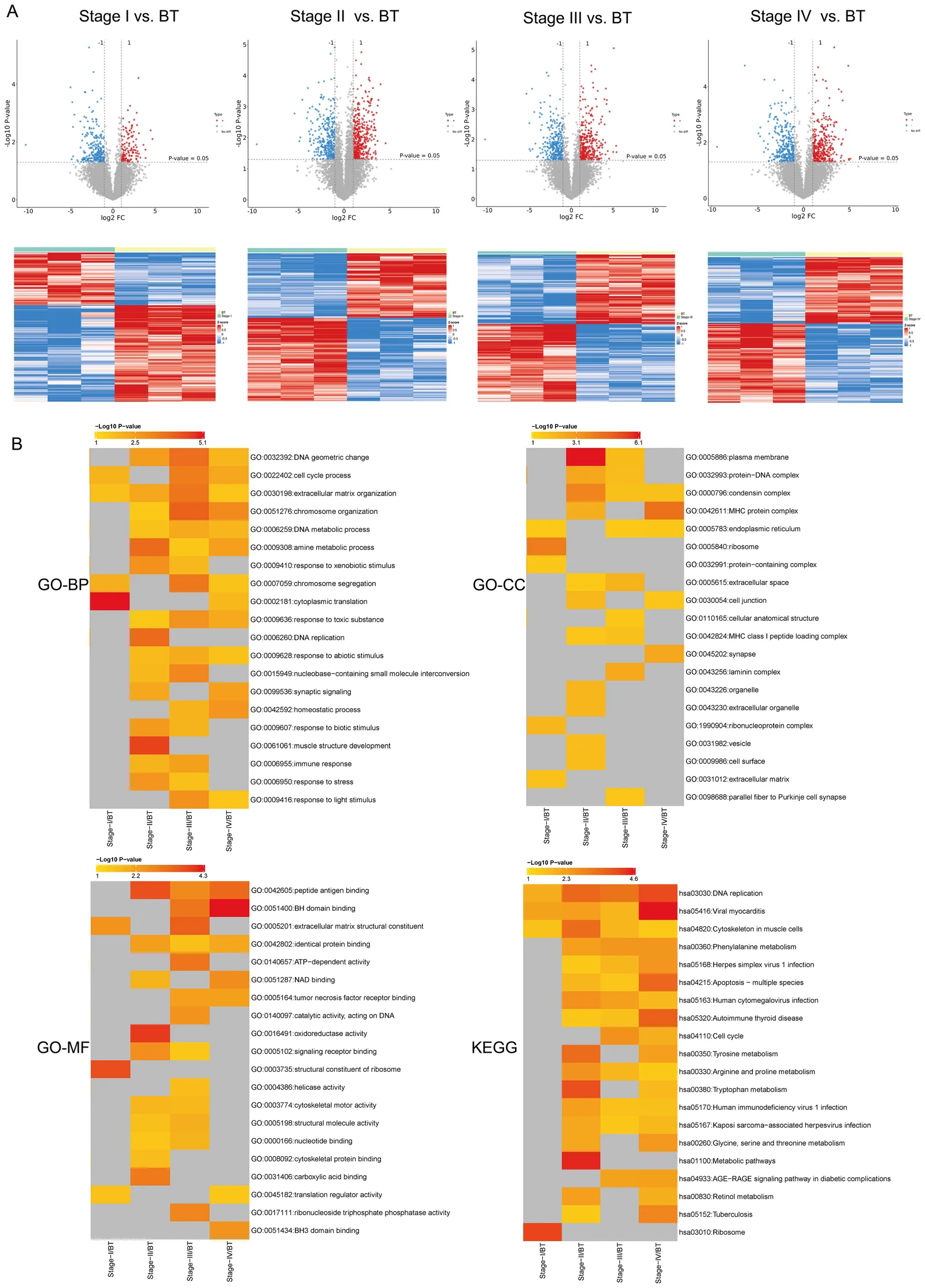
Stage-specific proteomic alterations and functional enrichment analysis. **(A)** Volcano plots (upper panel) and corresponding heatmaps (lower panel) display DAPs in each cancer stage vs. BT. Red means upregulated DAPs, and blue means downregulated DAPs. Heatmaps depict Z-score normalized expression of these stage-specific DAPs. **(B)** Functional characteristics of stage-specific DAPs with cellular component, biological process, molecular function, and KEGG pathway analyses.

DAPs in each stage of OVs compared to BT were visualized with volcano plots and heatmaps (**Figure 2A**). Functional enrichment analysis of stage-specific DAPs revealed distinct MFs and signaling pathways at different stages of OV, demonstrating progressive alterations during tumor development (**Figure 2B**; **Supplementary Table 3**).

#### 3.2.1 Stage I vs. BT (382 DAPs)

For biological processes (BPs), DAPs were significantly enriched in cytoplasmic translation, chromosome segregation, cell cycle process, and extracellular matrix (ECM) organization. For cellular components (CCs), DAPs were primarily enriched in the endoplasmic reticulum, ribosome, protein-containing complex, ribonucleoprotein complex, and ECM. For MFs, DAPs were significantly enriched in ribosome structural constituent, structural constituent of ECM, and translation regulator activity. For KEGG pathways, DAPs were significantly enriched in DNA replication, cytoskeleton in muscle cells, and ribosome.

#### 3.2.2 Stage II vs. BT (734 DAPs)

For BPs, DAPs were mainly enriched in muscle structure development, amine metabolic process, xenobiotic stimulus response, DNA replication, biotic stimulus response, and stress response. For CCs, DAPs were mainly enriched in the plasma membrane, protein-DNA complex, condensin complex, and MHC protein complex. For MFs, DAPs were significantly involved in peptide antigen binding, oxidoreductase activity, identical protein binding, signaling receptor binding, and carboxylic acid binding. For KEGG Pathways, DAPs were mainly enriched in DNA replication, cytoskeleton in muscle cells, phenylalanine metabolism, cell cycle, tyrosine metabolism, tryptophan metabolism, and metabolic pathways.

#### 3.2.3 Stage III vs. BT (649 DAPs)

For BPs, DAPs were mainly enriched in DNA geometric change, cell cycle process, ECM organization, chromosome organization, chromosome segregation, and nucleobase-containing small molecule interconversion. For CCs, DAPs were mainly localized in the extracellular space, and laminin complex. For MFs, DAPs were significantly enriched in peptide antigen binding, BH domain binding, ECM structural constituent, ATP-dependent activity, and ribonucleoside triphosphate phosphatase activity. For KEGG pathways, DAPs were mainly enriched in DNA replication, phenylalanine metabolism, and cell cycle.

#### 3.2.4 Stage IV vs. BT (659 DAPs)

For BPs, DAPs were significantly enriched in chromosome organization, DNA metabolic process, amine metabolic process, synaptic signaling, and homeostatic process. For CCs, DAPs were enriched in the condensin complex, MHC protein complex, endoplasmic reticulum, and synapse. For MFs, DAPs were mainly enriched in peptide antigen binding, BH domain binding, identical protein binding, tumor necrosis factor receptor binding, NAD binding, and BH3 domain binding. For KEGG pathways, DAPs were significantly enriched in DNA replication, apoptosis - multiple species, human cytomegalovirus infection, autoimmune thyroid disease, and cell cycle.

### 3.3 Functional, pathway, and subcellular localization analyses of All DAPs across different stages of OVs

**Figure 3:**
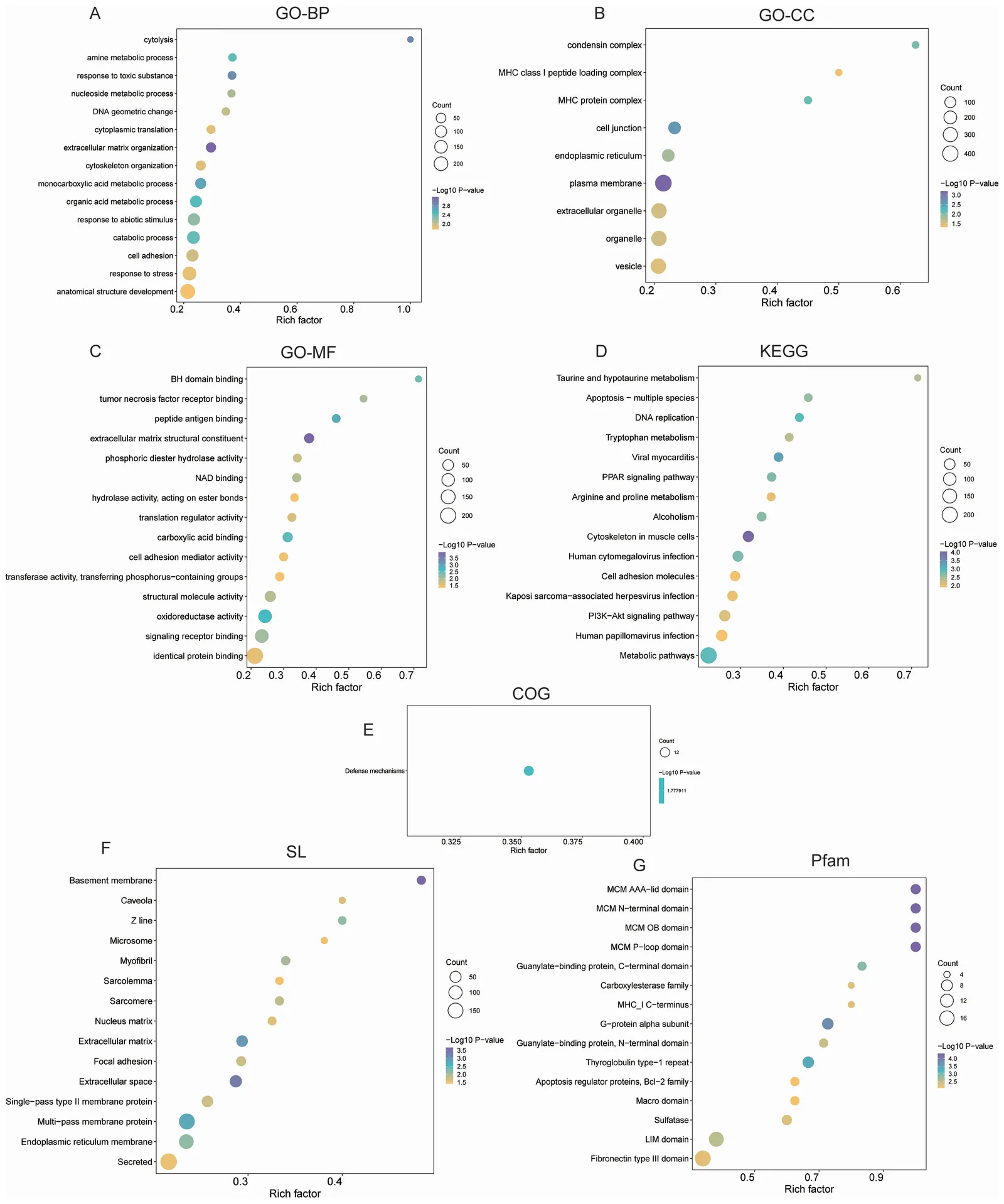
Functional characteristics of all DAPs across different stages of ovarian cancers. The 1,669 DAPs identified across all tumor stages were systematically annotated with BPs **(A)**, CCs **(B)**, MFs **(C)**, KEGG pathways **(D)**, COG classification **(E)**, subcellular localization **(F)**, and Pfam domain enrichment **(G)**. With up to the top 15 most significant results presented for each category. BPs, biological processes; CC, cellular components; MFs, Molecular functions.

The 1,669 DAPs across different stages of OVs compared to BT were subjected to comprehensive functional characterization (**Figure 3**; **Supplementary Table 4**). For BPs, DAPs were mainly enriched in cytolysis, DNA geometric change, cytoplasmic translation, ECM organization, and cell adhesion (**Figure 3A**). For CCs, DAPs were mainly localized in the condensin complex, MHC protein complex, MHC class I peptide loading complex, cell junction, and plasma membrane (**Figure 3B**). For MFs, DAPs were significantly enriched in BH domain binding, tumor necrosis factor receptor binding, peptide antigen binding, ECM structural constituent, signaling receptor binding, and identical protein binding (**Figure 3C**). For KEGG pathways, DAPs were mainly enriched in DNA replication, PPAR signaling pathway, PI3K-Akt signaling pathway, cell adhesion molecules, human papillomavirus infection, and metabolic pathways (**Figure 3D**). COG analysis of DAPs found defense mechanisms (**Figure 3E**). SL prediction found that DAPs were mainly distributed in the basement membrane, nuclear matrix, ECM, focal adhesion, endoplasmic reticulum membrane (multi-pass membrane protein), and secreted regions (**Figure 3F**). Pfam protein domain analysis of DAPs identified MCM N-terminal domain, MCM AAA-lid domain, MCM P-loop domain, MCM OB domain, MHC-I C-terminus, G-protein alpha subunit, and thyroglobulin type-1 repeat (**Figure 3G**).

### 3.4 Trends clustering patterns and pathways of OV proteins

**Figure 4:**
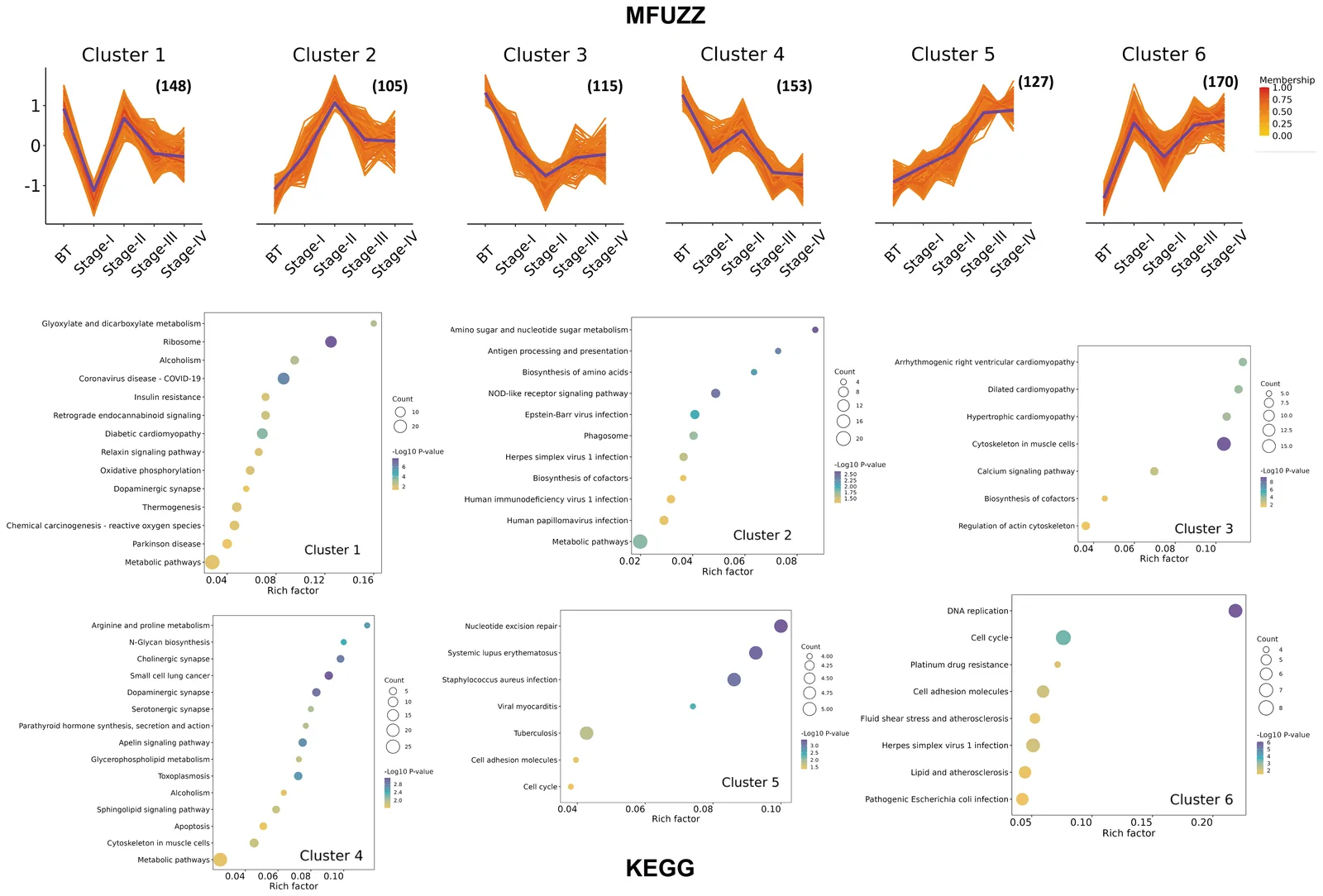
Trend cluster analysis of DAPs and pathways in each trend cluster, revealed by MFUZZ clustering analysis. These time-resolved trend patterns highlight proteins with stage-specific regulation, offering potential biomarkers and therapeutic targets for predictive and personalized medicine strategies.

The trend clustering analysis of 1,669 DAPs with MFUZZ software revealed six distinct trend clustering patterns across different stages of OVs. For MFUZZ analysis, proteins with a membership value > 0.5 were considered core cluster members, which yielded a set of 818 core proteins for downstream analysis (**Supplementary Table 5**). KEGG pathway analysis of the DAPs in each trend cluster elucidated the stage-aligned functional characteristics and biological processes of those DAPs in each trend cluster with potential relevance for personalized medicine (**Figure 4**; **Supplementary Table 6**).

#### 3.4.1 Trend cluster 1

A total of 148 proteins in this cluster showed highest expression in BT, lowest in stage I, rapid elevation in stage II, and a slight decline maintained at low levels in stages III and IV. KEGG pathway analysis found that DAPs were mainly involved in ribosome, relaxin signaling pathway, chemical carcinogenesis - reactive oxygen species (ROS), oxidative phosphorylation, and metabolic pathways. These proteins, activated and peaking in Stage II, represent potential predictive biomarkers for early diagnosis.

#### 3.4.2 Trend cluster 2

A total of 105 proteins in this cluster exhibited lowest expression in BT, continuously increased through stages I and II and peaked at stage II, followed by a slight decrease stabilizing in later stages. KEGG pathway analysis revealed those DAPs were mainly involved in amino sugar and nucleotide sugar metabolism, antigen processing and presentation, biosynthesis of amino acids, NOD-like receptor signaling pathway, Epstein-Barr virus infection, phagosome, biosynthesis of cofactors, and metabolic pathways. The enriched antigen processing and presentation and NOD-like receptor signaling pathway, combined with the expression pattern, suggests these proteins function in early immune escape mechanisms, making them suitable predictive markers for early diagnosis.

#### 3.4.3 Trend cluster 3

A total of 115 proteins in this cluster demonstrated high expression in BT and maintained consistently low expression across stages I-IV, with a slight decrease in stage II. KEGG pathway analysis revealed those DAPs were mainly involved cytoskeleton in muscle cells, calcium signaling pathway, and biosynthesis of cofactors.

#### 3.4.4 Trend cluster 4

A total of 153 proteins in this cluster displayed highest expression in BT, slightly decreased in stage I, modestly elevated in stage II, but showed an overall declining trend with disease progression. KEGG pathway analysis found that DAPs were mainly involved in arginine and proline metabolism, N-glycan biosynthesis, glycerophospholipid metabolism, sphingolipid signaling pathway, apoptosis, cytoskeleton in muscle cells, and metabolic pathways. The expression pattern in this trend cluster showed a strong negative correlation with disease progression, which combined with the enriched apoptosis activation and sphingolipid signaling involving pro-apoptotic ceramide (31)), it might be associated with tumor-suppressive functions, maintaining cellular homeostasis, inhibiting proliferation, and promoting cell death.

#### 3.4.5 Trend cluster 5

A total of 127 proteins in this cluster showed low expression in BT, elevated in stage I compared to BT, and progressively increased with cancer progression, stabilizing at high levels in stage IV. KEGG pathway analysis revealed that those DAPs were mainly involved in nucleotide excision repair, systemic lupus erythematosus, cell adhesion molecules, and cell cycle. Those proteins with gradual elevation with the increased stages might be associated with the accelerated proliferation, enhanced drug resistance through dysregulated cell cycle (32), potentially compromised platinum efficacy via enhanced DNA repair (33), and peritoneal metastasis through adhesion molecule overexpression (34).

#### 3.4.6 Trend cluster 6

A total of 170 proteins in this cluster demonstrated low expression in BT, high expression in stage I, slight decrease in stage II, and return to stable high expression in stages III and IV. KEGG pathway found that DAPs were mainly involved in DNA replication, cell cycle, platinum drug resistance, fluid shear stress and atherosclerosis, and cell adhesion molecules. These proteins potentially drive OV proliferation through DNA replication and cell cycle regulation (32), promote metastasis via adhesion molecule remodeling (34), and confer survival advantages in advanced tumors through lipid metabolic reprogramming (35) and platinum resistance (33).

### 3.5 PPI network-based hub molecules in OVs

**Figure 5:**
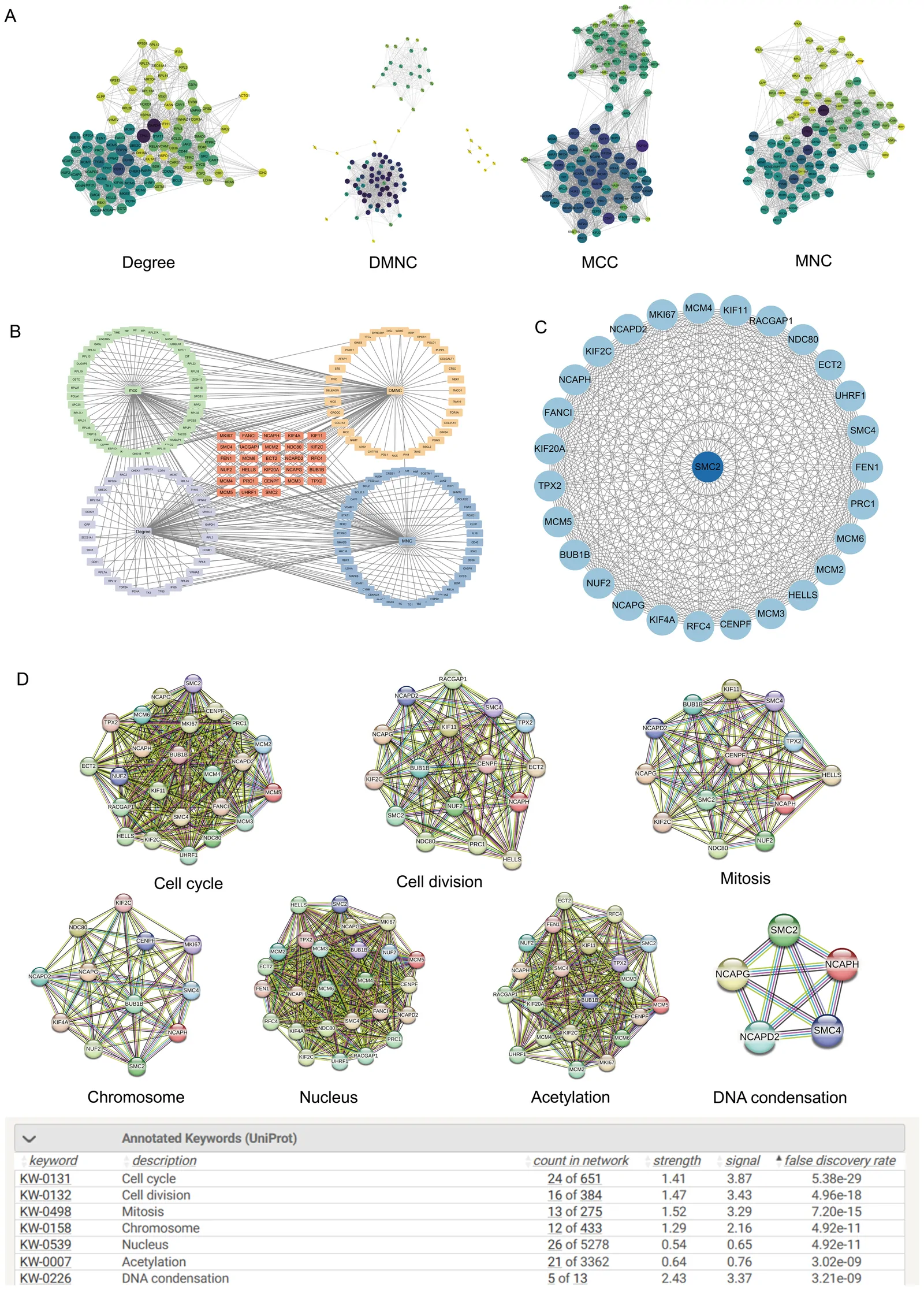
Protein-protein interaction (PPI) network analysis identifies SMC2 as a central hub. **(A)** PPI network analysis of 1,669DAPs and identification of hub genes with four topological algorithms (MNC, MCC, DMNC, and Degree). **(B)** Intersection of the top 100 hub genes from each algorithm identified 28 high-confidence hub genes. **(C)** PPI network of the 28 hub genes formed a highly interconnected module with 28 nodes and 369 edges. **(D)** Functional annotation of the 28 hub proteins with UniProt keywords found that these hub proteins were mainly involved in cell cycle, mitosis, chromosome-related, and nuclear processes.

PPI network analysis of the 1,669 DAPs revealed that 1,595 proteins were successfully mapped in the STRING database. Network topology was evaluated using four centrality algorithms (MCC, MNC, DMNC, and Degree) (**Figure 5A**; **Supplementary Table 7**). The top-100 hub genes from each algorithm were intersected, yielding 28 high-confidence hub genes that form a densely interconnected functional module (**Figure 5B**). This module comprises 28 nodes and 369 edges, indicating extensive PPIs (**Figure 5C**). The 28 hub proteins are: MK167, FANCI, NCAPH, KIF4A, KIF11, SMC4, RACGAP1, MCM2, NDC80, KIF2C, FEN1, MCM6, ECT2, NCAPD2, RFC4, NUF2, HELLS, KIF20A, NCAPG, BUB1B, MCM4, PRC1, CENPF, MCM3, TPX2, MCM5, UHRF1, and SMC2. Based on their MFs and reported roles in OV, these 28 hub proteins can be categorized into four functional groups:

#### 3.5.1 DNA replication and cell cycle progression

The MCM2-6 helicase complex (MCM2, MCM3, MCM4, MCM5, MCM6; minichromosome maintenance complex components 2–6) (36), the proliferation marker MK167(Ki-67), the DNA repair factor FEN1(flap endonuclease 1), and the mitotic regulator TPX2(targeting protein for Xklp2).

#### 3.5.2 Spindle assembly and chromosome segregation

Mitotic kinesins (KIF4A, KIF11, KIF2C, KIF20A), condensin components (SMC2, SMC4, NCAPH, NCAPD2, NCAPG), the NDC80 kinetochore complex (NDC80, NUF2), the spindle checkpoint kinase BUB1B, and cytokinesis regulators (PRC1, CENPF, RACGAP1).

#### 3.5.3 Epigenetic and transcriptional regulation

The chromatin remodeler HELLS and the epigenetic reader UHRF1 (ubiquitin like with PHD and ring finger domains 1).

#### 3.5.4 DNA repair and genome stability maintenance

The Fanconi anemia pathway protein FANCI, the replication factor C subunit RFC4, and the RhoGEF ECT2 (epithelial cell transforming 2).

Notably, SMC2 itself is a core component of the condensin complex, forming a heterodimer with SMC4 to drive chromosome condensation and segregation during mitosis. Functional annotation with UniProt keywords confirmed that these 28 hub proteins are predominantly involved in cell cycle, cell division, mitosis, chromosome organization, nuclear localization, acetylation, and DNA condensation (**Figure 5D**). Among them, 24 out of 28 hub proteins are directly linked to cell cycle regulation.

SMC2 emerges as a topologically central node within this network (**Figure 5C**), but the entire 28-protein module — rather than any single protein — likely functions as a coordinated unit integrating multiple layers of cell cycle control, from DNA replication and checkpoint activation to mitosis and cytokinesis, thereby acting as a core driver of OV cell proliferation and genomic instability. The identification of this module provides a rich resource for understanding stage-specific regulatory mechanisms and prioritizing targets for further functional studies.

### 3.6 Functional roles of SMC2 in OVs

**Figure 6:**
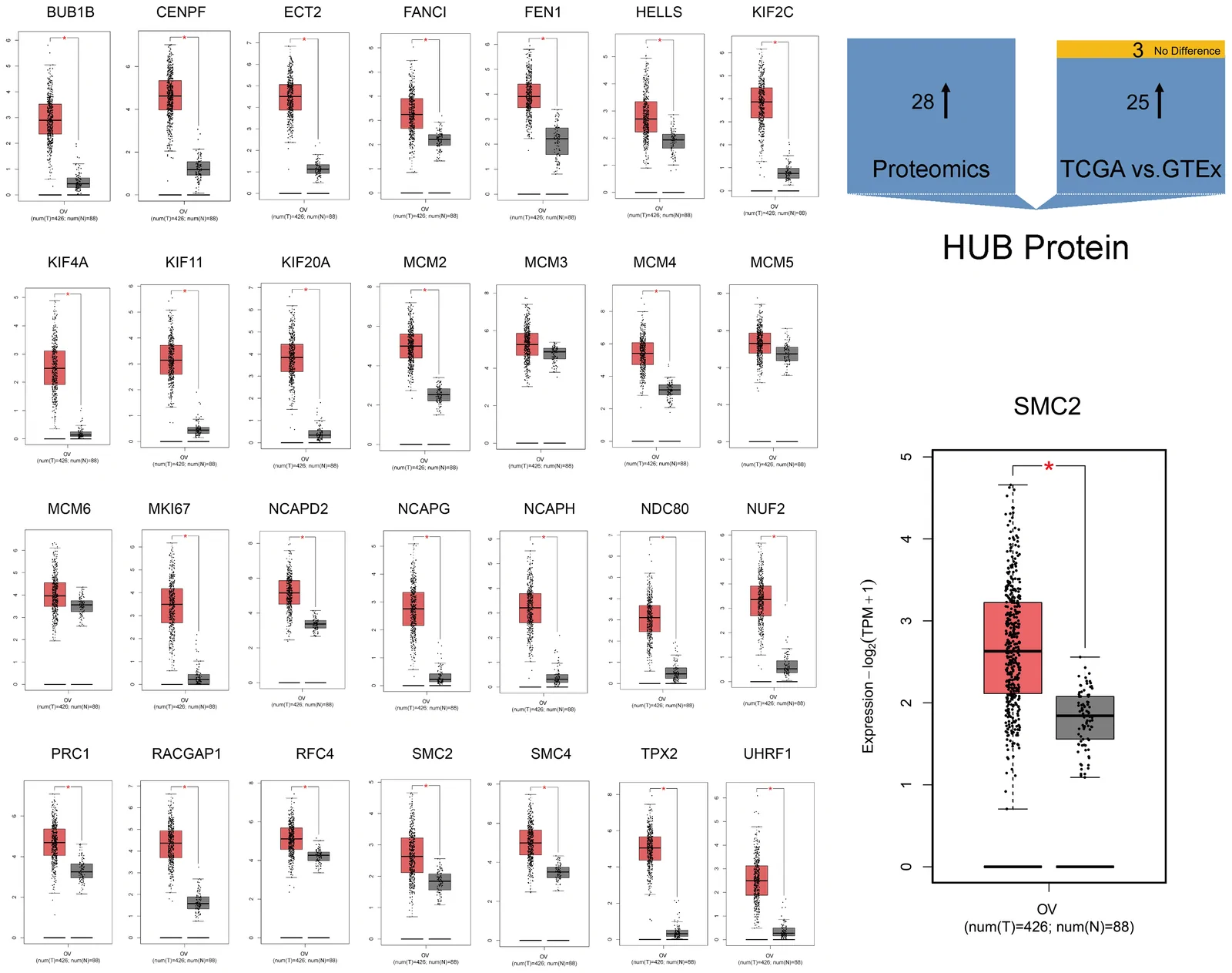
Transcriptomic validation of proteomic hub proteins. Transcriptomic analysis (TCGA vs. GTEx) confirms the upregulation of most identified hub proteins in ovarian cancers. Among 28 hub proteins, SMC2 shows significant overexpression (*p* < 0.01). Intersection analysis showed concordant upregulation at both RNA and protein levels for 25 proteins, while three (MCM3, MCM5, and MCM6) are discordant.

**Figure 7:**
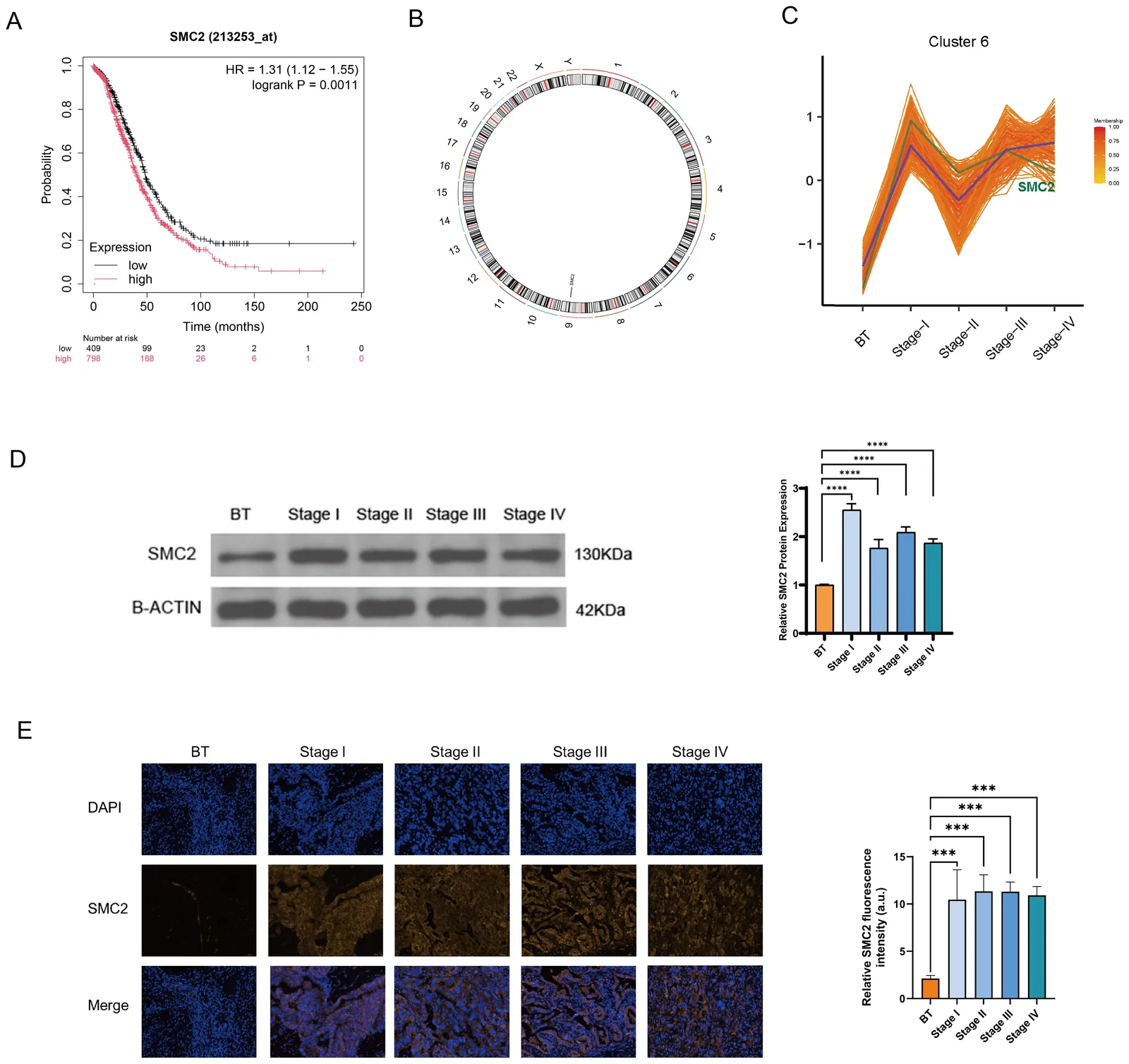
Validation of SMC2 in ovarian cancer. **(A)** Prognostic value: High *SMC2* mRNA expression correlates with worse OS (HR = 1.31, 95% CI: 1.12–1.55, *P* = 0.0011). **(B)** Schematic of the SMC2 genomic locus. **(C)** Trend expression pattern of SMC2 in MFUZZ cluster 6 (low in BT, peaks in stage I, sustained thereafter). **(D)** Western blot analysis confirms stage-dependent increase of SMC2 protein in ovarian cancer tissues. **(E)** mIHC validates SMC2 upregulation and nuclear localization in cancer tissues across stages (SMC2, red; DAPI, blue). Data are mean ± SD (n = 3). ***P<0.001.

Integrated analysis of GTEx and TCGA data via the GEPIA platform demonstrated concordance between transcriptional expression and our proteomic findings for 25 of 28 previously identified hub proteins. These 25 hubs showed a marked upward shift in tumor tissues, with high distribution density significantly deviating from normal tissue expression patterns; and only three proteins (MCM3, MCM5, and MCM6) showed no significant difference. This independent validation substantially reinforces the reliability of our proteomic dataset. Notably, SMC2 expression was significantly elevated in OV tissues relative to normal controls (p < 0.01). Box plot analysis further confirmed this overall upward expression shift and high distribution density in tumors, suggesting a crucial regulatory role for SMC2 in OV initiation and progression (**Figure 6**). Kaplan-Meier survival analysis found that high SMC2 mRNA expression was relative to significantly shortened OS in patients with serous OV (HR = 1.31, 95% CI: 1.12-1.55, p = 0.0011; **Figure 7A**). These findings demonstrated that SMC2 might promote malignant behavior potentially through accelerated tumor growth, dissemination, genomic instability, or development of therapy resistance.

Western blot and mIHC analyses of BTs and OV tissues across different stages (I-IV) demonstrated that SMC2, localized at chromosome 9 (**Figure 7B**) and showed a progressive increase with advancing tumor stage (**Figure 7C**), was significantly lowly expressed in benign ovarian tissues, and was significantly upregulated in OV tissues (**Figure 7D**). Notably, SMC2 exhibited marked expression increased as early as stage I, highly consistent with our proteomics findings (**Figure 7C**), which means that SMC2 was involved in early disease pathogenesis, supporting its potential value as an early molecular biomarker. Further, validation with mIHC (**Figure 7E**) revealed enhanced SMC2 fluorescence signals across all OV stages compared to controls, confirming the upregulation trend at the protein level. Nuclear counterstaining with DAPI (blue) provided spatial context, demonstrating predominant nuclear localization of SMC2. This subcellular distribution further supports its potential role in gene expression regulation and chromosome stability maintenance.

## 4 Discussion

In this study, we used high-resolution DIA quantitative proteomics to construct the comprehensive, stage-resolved proteomic atlas of OV, covering benign, early-stage (FIGO I-II), and advanced-stage (FIGO III-IV) tissues. We quantified 8,758 proteins and identified 1,669 stage-associated DAPs. Functional analysis revealed a clear evolutionary trajectory: early-stage DAPs were mainly enriched in proliferation-related processes such as cell cycle and DNA replication, whereas late-stage DAPs shifted toward ECM organization, cell adhesion, and apoptosis regulation. MFUZZ temporal clustering further resolved six distinct expression patterns (C1–C6), illustrating how protein modules are dynamically engaged during disease progression. Strikingly, PPI network analysis identified a 28-protein core hub module highly enriched for cell cycle, mitosis, and chromosome stability, with SMC2 positioned as a topologically central node. SMC2 exhibited significantly upregulated from stage I and persisting through all later stages — which was independently validated at the protein level by western blot and mIHC. Moreover, high SMC2 mRNA expression was significantly associated with shorter OSin serous OV patients (HR = 1.31, P = 0.0011). Together, these findings provide a valuable proteomic resource for understanding stage-specific molecular events and nominate SMC2 as a promising early diagnostic biomarker and potential therapeutic target within the framework of PPPM. Below we discuss the key findings in detail.

### 4.1 OV proteomic landscape changes across different pathological stages

Our cross-stage quantitative proteomic analysis delineates a comprehensive and dynamic proteomic landscape underlying OV progression. The sequential comparison of each stage against benign controls revealed not only a quantitative increase in proteomic disturbances—from 382 DAPs in stage I to over 650 DAPs in later stages—but also, more importantly, a qualitative shift in the biological processes driving the disease progression, e.g., an early predominance of proliferation-related pathways shifting to ECM remodeling, immunosuppression, and metabolic reprogramming in advanced stages. To our knowledge, this is one of the most systematic descriptions of stage-resolved proteomic alterations across full-spectrum OV. Clinically, these stage-specific protein changes directly inform early diagnostic markers, discrimination of indolent from aggressive tumors, and stage-adapted therapeutic targets. Moreover, the timing of qualitative pathway shifts may help guide clinical decisions—for instance, identifying which early-stage patients warrant more aggressive adjuvant therapy.

### 4.2 Activation of early proliferation-driving pathways and OV initiation

In stage I, the proteome was already markedly reconfigured, with significant enrichment in processes fundamental to rapid cell growth and division, including cytoplasmic translation, ribosome biogenesis, and early cell cycle events (e.g., chromosome segregation). This finding aligns well with the hypothesized cell origins of OV. HGSOC is thought to arise from the fallopian tube epithelium, where acquiring unlimited proliferative capacity is the primary event during malignant transformation. Enrichment of cytoplasmic translation points to a marked increase in protein synthesis—the material basis of cell proliferation—while activation of ribosome biogenesis further amplifies this effect. Notably, the enrichment of proteins involved in chromosome segregation and cell cycle progression indicates that aberrant mitotic regulation already exists at stage I, consistent with the clinical observation that OV exhibits high CIN from very early stages. The molecular machinery driving uncontrolled proliferation is established at the very onset of malignancy, a finding with important clinical implications. It suggests that interventions targeting cell cycle regulatory pathways could be effective at a very early stage of the disease, providing a theoretical basis for chemoprevention or early intervention in high-risk populations.

Progressing to stage II, the proteomic signatures further expand and diversify. DNA replication and metabolic pathways (e.g., amino acid metabolism) become significantly enhanced, and immune regulatory processes such as antigen presentation begin to emerge. The enrichment of DNA replication acts in concert with cell cycle activation to jointly fuel rapid tumor cell expansion. Activation of metabolic pathways reflects the early emergence of the Warburg effect, whereby tumor cells tend to rely on glycolysis rather than oxidative phosphorylation even under aerobic conditions. The enrichment of amino acid metabolism is particularly noteworthy, as various amino acids (e.g., glutamine) serve as essential nitrogen sources for proliferating tumor cells; metabolic reprogramming of amino acids has been established as a hallmark of many cancers.

The early involvement of immune regulatory processes such as antigen presentation at stage II has important implications for understanding how OV initially interacts with the immune system. Previous views held that OV develops an immunosuppressive microenvironment only at later stages. However, our finding that antigen processing and presentation-related proteins are already significantly altered at stage II suggests that immune recognition may be engaged early in tumor progression. This phenomenon likely reflects a “two-way game” between the tumor and the immune system: the tumor attempts to evade T-cell recognition through antigen presentation pathways, while the immune system tries to eliminate aberrant cells. The disruption of this dynamic balance may represent a critical juncture in disease progression.

### 4.3 Enrichment of microenvironment-remodeling pathways in advanced stages and metastatic mechanisms

At stages III and IV, pathways involved in tumor-microenvironment interactions become dominant. Differentially expressed proteins are significantly enriched in ECM organization, cell adhesion, and regulation of apoptosis, reflecting the tumor’s need for tissue invasion, cell survival, and adaptation to a complex microenvironment. Enrichment of ECM organization represents a key molecular basis for peritoneal metastasis in OV (37). OV typically disseminates via exfoliated cells implanting onto the peritoneal surface, a process that depends on degradation and remodeling of the ECM. Altered expression of ECM-related proteins (e.g., collagens, laminins, matrix metalloproteinases) directly affects tumor cell invasiveness and implantation efficiency. Enrichment of cell adhesion pathways reflects enhanced interaction between tumor cells and peritoneal mesothelial cells, a critical step in establishing peritoneal implants.

The enrichment of extracellular vesicle pathways (38) is a notable new finding. Recent studies have shown that tumor-derived extracellular vesicles carry various bioactive molecules (including proteins, nucleic acids, and lipids) and play key roles in tumor microenvironment remodeling, immune modulation, and pre-metastatic niche formation. Extracellular vesicles secreted by OV cells can be taken up by peritoneal mesothelial cells, promoting mesothelial-to-mesenchymal transition and creating a “soil” for tumor implantation. The enrichment of extracellular vesicle-related proteins in this study offers a new perspective on the molecular mechanisms underlying peritoneal metastasis of OV.

Enrichment of apoptosis regulation pathways (39) suggests that advanced tumors acquire enhanced anti-apoptotic capacity. Tumor cells evade chemotherapy-induced cell death by upregulating anti-apoptotic proteins (e.g., BCL-2 family members) and downregulating pro-apoptotic proteins (e.g., BAX, BAD). This finding aligns well with the clinical observation that sensitivity to platinum-based chemotherapy declines as OV progresses, eventually leading to acquired resistance.

### 4.4 Biological significance and clinical implications of the functional shift

The functional shift from a proliferation-driven program in early stages to microenvironment-remodeling in advanced stages reveals the dynamic evolution of core biological needs during OV progression. In early disease, the primary task of tumor cells is to establish a proliferative advantage, achieving rapid expansion by activating pathways such as cell cycle, DNA replication, and protein synthesis. Once the tumor reaches a certain size and the local microenvironment can no longer support its growth, tumor cells begin to invade surrounding tissues and remodel the microenvironment to meet their survival needs. This shift resembles the “proliferation-to-invasion switch” observed in several solid tumors.

Of note, this functional shift is not a simple on-off switch but rather a gradual process. Stage II serves as a transitional phase, simultaneously exhibiting activation of both proliferation-related and microenvironment-related pathways, suggesting a “window period” during which molecular events accumulate and functional transformation occurs. Identifying key molecular events within this window period holds important implications for developing intervention strategies aimed at blocking disease progression.

### 4.5 Coordinated regulation of nuclear and extracellular functional modules: a global functional characteristic of OV progression

Beyond stage-specific changes, this study systematically analyzed the global functional features of all 1,669 differentially expressed proteins, revealing a coordinated regulatory network of two core functional modules during OV progression.

Functional enrichment analysis showed that the differentially expressed proteins were mainly concentrated in two functional modules: an “intranuclear module” associated with cell cycle and chromosome stability, and an “extracellular module” associated with ECM and microenvironment remodeling. At the biological process level, the differentially expressed proteins were significantly enriched in cell cycle-related processes such as DNA geometric alteration and cytoplasmic translation, as well as microenvironment-related processes including ECM organization and cell adhesion. This dual enrichment pattern of “intranuclear-extracellular” suggests that OV progression may depend simultaneously on rapid proliferation and invasive dissemination.

CC analysis further supported this dual-module classification. For the intranuclear module, the differentially expressed proteins were mainly localized to the condensin complex, MHC protein complex, and ribonucleoprotein complex. Enrichment of the condensin complex—composed of SMC2, SMC4, and NCAP family proteins—is particularly noteworthy, as this complex directly governs chromosome condensation and segregation. For the extracellular module, the differentially expressed proteins localized to the basement membrane, ECM, focal adhesion, and cell-cell junctions, structures that serve as critical interfaces for tumor-microenvironment interactions.

At the MF level, the differentially expressed proteins were significantly enriched in immune-related functions such as BH domain binding, tumor necrosis factor receptor binding, and peptide antigen binding, as well as microenvironment-interaction functions including ECM structural constituent and signaling receptor binding. These features indicate that OV progression involves not only cell cycle dysregulation and microenvironment remodeling but also active participation of immune regulation—consistent with the finding that antigen presentation-related proteins are already enriched at stage II.

KEGG pathway enrichment analysis revealed more specific molecular mechanisms. Enrichment of DNA replication and cell cycle pathways formed the core of the intranuclear module, whereas enrichment of the PPAR signaling pathway, PI3K-Akt signaling pathway, and cell adhesion molecules constituted the core of the extracellular module. The PPAR signaling pathway is involved in lipid metabolism and inflammatory regulation, and its activation is closely linked to tumor metabolic reprogramming (40, 41). The PI3K-Akt signaling pathway is a central regulator of cell survival, proliferation, and migration; its aberrant activation has been shown to be associated with invasion, metastasis, and chemotherapy resistance in various tumors (42). The cell adhesion molecule pathway directly mediates interactions between tumor cells and peritoneal mesothelial cells, a key step in peritoneal implantation metastasis of OV (37, 43).

Pfam analysis identified multiple domains of the MCM family (MCM2-7), including the N-terminal domain, AAA-lid domain, P-loop domain, and OB domain. This family is a core component of the DNA replication helicase, and its enrichment further reinforces the central role of DNA replication and the cell cycle in OV (44, 45). Enrichment of the MHC-I C-terminal domain echoes the antigen processing and presentation pathway, suggesting that immune recognition may persist during OV progression (46).

SL prediction showed that the differentially expressed proteins were mainly distributed in the basement membrane, nuclear matrix, ECM, focal adhesion, endoplasmic reticulum membrane, and secretory regions. This distribution pattern suggests that malignant transformation of OV cells involves not only abnormalities in nuclear gene expression and DNA replication regulation but also systemic changes in cell-microenvironment interactions—from intranuclear transcriptional regulation and DNA replication to signal reception and adhesive junctions at the membrane, and further to ECM remodeling and exosome secretion—forming a multi-layered network of malignant transformation.

In summary, the global functional analysis of 1,669 differentially expressed proteins reveals coordinated regulation of an “intranuclear functional module” (cell cycle, DNA replication, chromosome condensation) and an “extracellular functional module” (ECM, cell adhesion, extracellular vesicles) during OV progression. This finding is highly consistent with the stage-specific analysis (proliferation-driven in early stages, microenvironment-remodeling in advanced stages), together constructing a functional landscape of malignant transformation in OV from both global and stage-resolved perspectives. The coordinated action of these functional modules provides a functional foundation for subsequent PPI network analysis and identification of SMC2 as a hub protein.

### 4.6 Dynamic interpretation of differential protein expression trends and functional module analysis revealed by MFUZZ clustering analysis

MFUZZ clustering analysis transformed static stage-wise comparisons into a dynamic model, revealing six coherent protein expression trajectories (C1–C6)贯穿 disease progression. The coordinated expression dynamics of proteins within each cluster suggest that they may function as modules at specific pathological stages. This soft clustering method allows proteins to belong to multiple clusters with varying degrees of membership, more faithfully reflecting the complexity and overlap of MFs in biological systems, and is particularly suitable for analyzing continuous, dynamic pathological processes such as tumor progression.

(i) Clusters C1 and C2, which peaked at early stages, were enriched in metabolic and immune regulation pathways, suggesting their involvement in early metabolic reprogramming and immune interactions. Cluster C1 showed high expression in benign tissues, a drop to the lowest level at stage I, and a rapid rebound by stage II. This “V-shaped” trajectory implies that these proteins may undergo transient suppression during OV initiation and then recover to exert functions at stage II. The enriched relaxin signaling pathway and oxidative phosphorylation pathway in this cluster warrant further investigation. Relaxin is a peptide hormone with anti-fibrotic and vasodilatory effects and has recently been implicated in tumor microenvironment remodeling; enrichment of oxidative phosphorylation suggests adaptive adjustments in mitochondrial function at stage II. Cluster C2 exhibited a steadily increasing expression pattern, peaking at stage II and then stabilizing. This trajectory aligns with immune response activation—enrichment of antigen processing and presentation and NOD-like receptor signaling pathways indicates early immune recognition and initiation of inflammatory responses (47). Its marked stage-specific expression makes this cluster a candidate for exploring early warning signals, though this requires further study.

In contrast, clusters C5 and C6 showed progressive upregulation and were closely associated with DNA replication, cell cycle, and platinum-drug resistance pathways. This association echoes the clinical phenotype of increased tumor aggressiveness and treatment failure in advanced stages, suggesting that these proteins may contribute to such adverse features. Cluster C5 exhibited a gradual upregulation trajectory (from low expression in benign tissues to stable high expression at stage IV), highly consistent with the clinical observation that tumor malignancy increases with advancing stage. The enriched cell cycle and cell adhesion molecule pathways in this cluster jointly drive rapid tumor proliferation and dissemination. Particularly noteworthy is the enrichment of the nucleotide excision repair pathway—the main repair pathway for DNA damage induced by platinum-based chemotherapy, and its enhanced activity is directly linked to platinum resistance. This association echoes the clinical phenotype of treatment failure and poor prognosis in advanced stages, suggesting that C5 cluster proteins may constitute a “poor-prognosis signature” module.

Cluster C6 displayed a unique trajectory: high expression at stage I, a moderate decrease at stage II, and a return to high expression at stages III-IV. The biological significance of this expression pattern merits further exploration. High expression at stage I reflects the urgent need for cell cycle drive at the onset of malignant transformation, with SMC2—a core subunit of the condensin complex—being rapidly activated to support chromosome condensation and cell division. The slight decrease at stage II may represent a functional transition window from *in situ* growth to local invasion, during which tumor cells adjust their molecular programs to adapt to new microenvironmental demands, leading to a transient adjustment of cell cycle-related proteins. At stages III-IV, as the tumor enters a phase of rapid progression, the demand for cell cycle drive intensifies again and C6 cluster proteins return to high expression. Concurrently, tumor heterogeneity may also contribute to sample-to-sample variability, but this fluctuation does not alter the core pattern of early activation and sustained high expression.

Cluster C4 showed decreasing expression with disease progression and was enriched in pro-apoptotic and tumor-suppressive functions. Its negative correlation with disease stage suggests a gradual loss of cellular protective mechanisms during malignant progression. The enrichment of the sphingolipid signaling pathway in this cluster (31, 48) is particularly noteworthy. Sphingolipids are bioactive lipid molecules, among which ceramide has been established as a pro-apoptotic second messenger that induces tumor cell death by activating the mitochondrial apoptotic pathway. The decline of C4 cluster proteins with disease progression may indicate that advanced tumor cells acquire anti-apoptotic capacity by downregulating pro-apoptotic signaling pathways, a speculation consistent with clinically observed chemotherapy resistance. Enrichment of N-glycan biosynthesis and glycerophospholipid metabolism further suggests that lipid metabolic reprogramming plays an important role in tumor progression, though this hypothesis awaits experimental validation.

Strikingly, cell cycle and genome stability pathways remained a central theme across all stages. This observation pervades the entire proteomic analysis—from stage-specific enrichments (cell cycle pathways enriched in both stage I and stage III), to global functional analysis (overall enrichment of DNA replication and cell cycle among differentially expressed proteins), to trend clustering analysis (both C5 and C6 clusters associated with the cell cycle)—suggesting that dysregulated cell cycle control serves as the “engine” of OV initiation and progression. Genome stability is a prerequisite for normal cell cycle progression, and processes such as chromosome segregation, DNA replication, and DNA repair together constitute a “protective network” that maintains genome stability.

OV, particularly the high-grade serous subtype, is characterized by profound CIN, with over 90% of patients harboring TP53 mutations and approximately 50% exhibiting homologous recombination repair deficiency. The persistent enrichment of cell cycle- and genome stability-related proteins in this study aligns well with these known molecular features, validating the core pathological characteristics of OV at the proteomic level. More importantly, this finding suggests that targeting cell cycle and DNA repair pathways may have broad therapeutic implications, extending beyond BRCA-mutant patients to a wider OV population.

This observation is concretely reflected in the PPInetwork analysis, which further identified a 28-protein core module centered on SMC2 that acts cooperatively in cell cycle regulation.

### 4.7 The 28-protein core module: a cooperative network regulating cell cycle and genome stability

PPI network analysis identified a core module of 28 highly interconnected proteins from the 1,669 differentially expressed proteins. These 28 proteins form a dense interaction network with 28 nodes and 369 edges, suggesting that they act in a highly coordinated manner rather than independently. Functional annotation shows that the majority are involved in cell cycle regulation, mitosis, and chromosome condensation, collectively covering multiple steps from DNA replication to cytokinesis. From a network biology perspective, this high internal connectivity indicates that the module executes a relatively independent and core biological function—driving cell cycle progression and maintaining genome stability. Perturbation of any single node may propagate through the network to affect the entire module. SMC2, as a topological hub of this network, when aberrantly expressed could thus disrupt the coordinated function of the whole module, impairing cell cycle regulation and genome stability. The topological structure of this module carries important biological implications. The 28 proteins form a dense interaction network (each node connects directly to ~13 other nodes on average), suggesting that these proteins act in a highly coordinated manner rather than independently. From a network biology perspective, high internal connectivity usually indicates that the module executes a relatively independent and core biological function—namely, driving cell cycle progression and maintaining genome stability. Perturbation of any single node may propagate through the network to affect the entire module, implying that SMC2, as a topological hub of this network, when aberrantly expressed could disrupt the coordinated function of the whole module, thereby affecting cell cycle regulation and genome stability.

From a translational perspective, identification of this module provides multiple candidate entry points for targeted therapy of OV. The MCM2-6 complex is upregulated in OV and associated with poor prognosis (49–51). KIF11 is a mitotic kinesin upregulated in OV; its overexpression not only predicts worse patient survival but is also closely associated with resistance to MAPK pathway inhibitors (52). KIF20A overexpression correlates with poorer progression-free survival and OS in ovarian clear cell carcinoma (53); KIF20A has been found to induce M2 macrophage polarization in OV, and its depletion modulates PTEN, enhances radiosensitivity, and suppresses OV development (54). Multiple subunits of the condensin complex (NCAPG, NCAPH) are highly expressed in OV and closely linked to poor prognosis (55, 56); among them, NCAPG promotes OV cell proliferation, migration, and invasion by activating the Akt signaling pathway, and downregulation of NCAPG inhibits OC cell proliferation and invasion by activating the p38 MAPK signaling pathway (57). The NDC80 complex (NDC80, NUF2) is highly expressed in OV and associated with poor prognosis and immune infiltration (58); NUF2 promotes EOC progression via the ERBB3-mediated PI3K-AKT and MAPK signaling axes (59); NUF2 is significantly elevated in OV cells, and knockdown of NUF2 markedly inhibits OV cell proliferation and invasion while promoting apoptosis and ferroptosis (60). BUB1B promotes OV cell proliferation and metastasis by activating the Wnt/β-catenin pathway (61). PRC1 participates in the maintenance of the stem cell compartment by silencing tumor suppressor genes and regulating stem cells; it consists of multiple Polycomb group (PcG) proteins that play roles in normal development and, when dysregulated, promote cancer progression (62); PRC1 is overexpressed in HGSOC and promotes disease progression by binding RPL4 and increasing MDM2-mediated p53 ubiquitination (63). CENPF is highly expressed in serous OV, and its knockdown significantly inhibits cell proliferation, migration, and invasion (64). UHRF1 is highly expressed in OV and promotes tumor progression through metabolic reprogramming and angiogenesis (65). FANCI is highly expressed in OV and correlates with poor survival and chemotherapy resistance; knockdown of FANCI inhibits cell migration and invasion (66–68). RFC4 has been identified as a potential biomarker in OV and pan-cancer (69–71), and is associated with poor prognosis and chemotherapy resistance in multiple tumor types. ECT2 copy number variation and protein expression correlate with chemotherapy response in serous OV and is one of the poor-prognosis genes in HGSOC (72, 73). RACGAP1 expression is elevated in OV, closely associated with clinicopathological features and patient prognosis, and can synergize with chemotherapeutic drugs to enhance anti-tumor efficacy (74). KIF4A is significantly upregulated in OV clinical samples, and its high expression is significantly associated with reduced OS. KIF2C is highly expressed in serous OV, significantly associated with poor prognosis, and promotes drug resistance and glycolysis in OV cells by upregulating PKM2 (75)(2024.129). TPX2 silencing inhibits OV cell proliferation *in vitro* and *in vivo* by increasing reactive oxygen species (ROS) levels and inducing apoptosis (76). TPX2 is one of the most powerful prognostic biomarkers in serous EOC, significantly associated with shorter OS and reduced disease-free survival (77). However, previous studies have mostly focused on individual molecules. In contrast, the present study finds that these molecules form a highly synergistic interaction network in OV, suggesting that combining targeting of multiple nodes within the module (e.g., simultaneous inhibition of DNA replication and spindle assembly) may produce synergistic anti-tumor effects and reduce the risk of single-agent resistance. This “module-targeting” strategy merits systematic evaluation in future studies.

Furthermore, many proteins in this module have been independently reported as potential prognostic markers in OV, but a systematic understanding integrating them into a network module has been lacking. This study, through PPI network analysis, reveals that these proteins have highly synergistic interactions in OV, suggesting that their co-expression and functional cooperation may be important drivers of the malignant phenotype in OV. This finding provides a new perspective for understanding the molecular pathology of OV and lays a foundation for developing module-based prognostic models and therapeutic strategies.

### 4.8 Value of the proteomic landscape within the PPPM framework

In summary, this multi-layered proteomic landscape provides a powerful resource for the PPPM framework: it offers (i) predictive insights—through stage-specific protein signatures that mark disease initiation and progression; (ii) preventive targets—by identifying key pathways whose intervention may block disease progression. Pathways such as cell cycle, DNA replication, and chromosome segregation are already significantly activated at stage I, suggesting that interventions targeting these pathways could be effective at a very early stage of the disease. Microenvironment-remodeling pathways dominate in advanced stages, indicating that late-stage interventions need to also address tumor microenvironment regulation; and (iii) a foundation for personalized medicine—by cataloging a large number of candidate biomarkers and therapeutic targets according to disease stage, among which SMC2 is a representative example emerging from this systematic analysis.

### 4.9 Biological significance of SMC2 in OV

SMC2, a member of the SMC family (78), forms a heterodimer with SMC4 during mitosis and is essential for chromosome condensation and segregation control. Accumulating evidence demonstrates aberrant SMC2 expression in various cancers, where its overexpression correlates strongly with aggressive tumor behavior and unfavorable prognosis. SMC2 depletion induces G2/M phase arrest, suppresses proliferation, promotes apoptosis, and impairs colony-forming ability,; xenograft models further demonstrate that SMC2 knockdown markedly inhibits tumor growth, highlighting its translational potential as a therapeutic target (79), SMC2 also contributes to CIN (80), and its overexpression is related to cancer poor prognosis (81, 82). Also, SMC2 knockdown suppresses malignant progression by upregulating BTG2 expression (83). SMC2 was a critical gene for mucinous EOC (mEOC) cell survival in CRISPR/Cas9 screens (84), and has been proposed as a potential target for CSCs, where nanocarrier-delivered anti-SMC2 antibodies exert significant antitumor effects alone or in combination with chemotherapy (85). Nevertheless, the role and dynamics of SMC2 in HGSOC, particularly across different disease stages, remain largely unexplored.

One of the most critical findings of this study is the revelation of unique dynamic expression pattern of SMC2 during OV progression. MFUZZ software clustering analysis assigned SMC2 to trend cluster 6, which was functionally enriched in pathways DNA replication, cell cycle, and platinum drug resistance. This cluster was characterized by its lowest expression in benign ovarian tissues, significant upregulation as early as stage I, and sustained high-level expression throughout subsequent stages (II-IV). This expression trajectory (**Figure 7C**) positions SMC2 as an early and persistently activated molecule in ovarian carcinogenesis. This characteristic distinguishes SMC2 from many other molecules that alter significantly only in advanced stages, endowing it with ideal features for an early warning biomarker. These results align strongly with SMC2-related biological function as follows: as a core component of the condensin complex, SMC2 is directly responsible for proper chromosome condensation and segregation during mitosis, making it a key molecule for maintaining genomic stability and regulating cell cycle progression (particularly M-phase) (79, 80). The early upregulation and sustained high expression of SMC2 in OV may drive malignant progression, clonal evolution, and therapy resistance by compromising chromosome segregation fidelity. This proposed role of SMC2 is consistent with reported oncogenic functions and associations with poor prognosis in various other cancers (79–83).

This study provides multi-layered evidence supporting SMC2 as a novel biomarker for OV: (i) Stage specificity and early onset: the significant upregulation of SMC2 in stage I is its core advantage as an early diagnostic marker, superior to traditional markers like CA125. (ii) Persistence and generality: The high expression state of SMC2 persists from stages I to IV, indicating its potential utility not only for early diagnosis but also for monitoring disease progression. (iii) Prognostic value: Kaplan-Meier survival analysis clearly demonstrated that high SMC2 mRNA expression was significantly associated with shortened OS in patients with serous OV (HR = 1.31, P = 0.0011). This aligns with the role of SMC2 in promoting malignant behaviors such as proliferation, metastasis, genomic instability, and drug resistance, strongly supporting high SMC2 expression as an independent risk factor for poor prognosis of OV patients, thereby holding significant prognostic assessment value. (iv) Reliability of tissue validation: WB and mIHC results perfectly corroborated the proteomics and transcriptomics (GEPIA) findings at the protein level, confirming the authenticity and detectability of SMC2 upregulation in OV tissues, particularly in the nucleus.

Moreover, integrated with the PPPM concept, SMC2 detection could be applied for risk stratification, early auxiliary diagnosis, and prognostic prediction.

This study not only emphasizes the diagnostic and prognostic value of SMC2 but also provides a strong rationale for its role as a therapeutic target. Several previous studies have suggested the anti-tumor potential of targeting SMC2 because its knockdown induced G2/M phase arrest, suppressed proliferation, promoted apoptosis, and inhibited *in vivo* tumorigenesis (79); SMC2 was identified as an essential gene for survival in mucinous OV cells via CRISPR/Cas9 screening (84); moreover, studies have directly proposed that targeting SMC2 (e.g., using nanocarriers to deliver anti-SMC2 antibodies) can effectively inhibit tumor growth, particularly against CSCs, and synergize with chemotherapy (85). Our study further confirm the crucial role and prevalent high expression of SMC2 in HGSOC, significantly expanding its applicability as a therapeutic target.

### 4.10 Innovation, limitations, and future directions

#### 4.10.1 Innovation

This study represents one of the first to systematically delineate the dynamic proteomic landscape across the complete disease stages of OV, from benign ovarian tissues to early and advanced stages, utilizing high-resolution DIA quantitative proteomics. We identified a 28-protein core module governing cell cycle and genomic stability, providing a network-level perspective on OV progression. We also identified and validated the significant upregulation of SMC2 as early as stage I, revealing its unique early activation and sustained high-expression pattern, and providing a promising new target for early diagnosis. By integrating multi-omics analyses (proteomics, transcriptomics), functional enrichment, PPI networks, survival analysis, and tissue validation, this study established a comprehensive and mutually corroborative evidence chain for the biological function, clinical significance, and translational value of SMC2 in OV at the proteomic and transcriptomic levels. Framing the discovery of SMC2 within the PPPM/3PM concept underscores its potential application in early disease prediction, risk stratification, and individualized intervention, encompassing both diagnostics and targeted therapy.

#### 4.10.2 Limitations

Although our study encompassed all stages of OVs, the sample size for stage I remained relatively small. Regarding tissue origin, this study focused on tissue proteomics. Whether SMC2, as a nuclear protein, can be stably released into the peripheral circulation and detected reliably (e.g., as a serum/plasma biomarker) remains unexplored. In terms of mechanistic depth, while our bioinformatic analysis and experimental validation (WB/mIHC) clarified expression pattern and localization of SMC2 and inferred its function based on enrichment analysis and literature, the specific molecular mechanisms by which SMC2 contributes to ovarian carcinogenesis and progression (particularly early initiation)—such as its downstream effectors and its role in CIN remain unexplored. Concerning subtype specificity, this study was limited to HGSOC, and other OV subtypes (e.g., mucinous, endometrioid, and clear cell) were not examined. Finally, the relationship between SMC2 expression levels and patient response to chemotherapy (especially platinum-based) or targeted therapies was not analyzed.

#### 4.10.3 Future directions

Future research directions include the following: (i) Large-sample validation: Validate the value of SMC2 protein expression for early diagnosis and prognosis in independent, large-scale, multi-center prospective cohorts (including sufficient early-stage samples) using standardized methods (e.g., IHC). (ii) Liquid biopsy biomarker development: Explore detection methods and clinical application potential for SMC2 and its derivatives (e.g., circulating nucleic acids, exosomal proteins) in blood, ascites, and other body fluids, aiming for non-invasive or minimally invasive diagnosis/monitoring. (iii) Molecular mechanism elucidation: Utilize OV cell lines, organoids, and animal models combined with genetic manipulation (e.g., CRISPR/Cas9, siRNA/shRNA) to elucidate SMC2 specific role in driving OV, particularly early lesions, focusing on its effects on cell cycle checkpoints, chromosomal stability, DNA damage repair, and stem cell properties. (iv) Integrated diagnostic model: Integrate SMC2 with existing biomarkers (CA125, HE4), imaging features, and gene mutation profiles to construct a more accurate multimodal model for early diagnosis, prognosis prediction, and therapy response prediction in OV. (v) Subtype analysis and therapy response prediction: Investigate the expression and functional differences of SMC2 across different OV subtypes and analyze the association between its expression levels and therapy sensitivity/resistance to guide individualized treatment. (vi) Targeted therapy investigation: Evaluate the efficacy of SMC2-targeting strategies [e.g., inhibitors, antibody-drug conjugates (ADCs), siRNA delivery systems] in OV models (e.g., organoids) and explore their synergistic effects with existing therapies (chemotherapy, PARPi, anti-angiogenic drugs).

## 5 Conclusion

This study established the comprehensive stage-resolved proteomic atlas of OV, covering benign, early (I–II) and advanced (III–IV) stages, using DIA quantitative proteomics. A total of 1,669 stage-associated DAPs were identified, revealing a functional shift from early proliferation-driven pathways (cell cycle, DNA replication) to late-stage microenvironment remodeling (ECM organization, cell adhesion, apoptosis). MFUZZ clustering resolved six dynamic expression trajectories (C1–C6), illustrating how protein modules are temporally engaged during disease progression. PPI network analysis further uncovered a 28-protein core module highly enriched for cell cycle, mitosis, and genome stability, with SMC2 as one of its central hub proteins. SMC2 exhibited an “early-activated, sustained high-expression” pattern (upregulated from stage I and persisting through all later stages), validated by Western blot and mIHC. High SMC2 mRNA expression was significantly associated with shorter OS (HR = 1.31, P = 0.0011). These findings not only provide a comprehensive proteomic resource for understanding stage-specific molecular events but also nominate the 28-protein module as a key cooperative network and SMC2 as a promising early diagnostic biomarker and therapeutic target within the PPPM framework. This study opens new avenues for early warning, precise diagnosis, prognostic assessment, and individualized therapy in OV.

## Data Availability

The original contributions presented in the study are included in the article/**Supplementary Material**. Further inquiries can be directed to the corresponding authors.
