# Supplementary material for: Ovarian cancer proteomic landscape changes across different pathological stages and biomarker-sets identified with integrative multiomics analysis
Source: Front Endocrinol (Lausanne). 2026 Sep 7;17:1898210. doi: 10.3389/fendo.2026.1898210 (PMC13590318; doi:10.3389/fendo.2026.1898210)

Supplementary Figure 1. the full uncropped Gels and Blots image(s) for Figure 7D

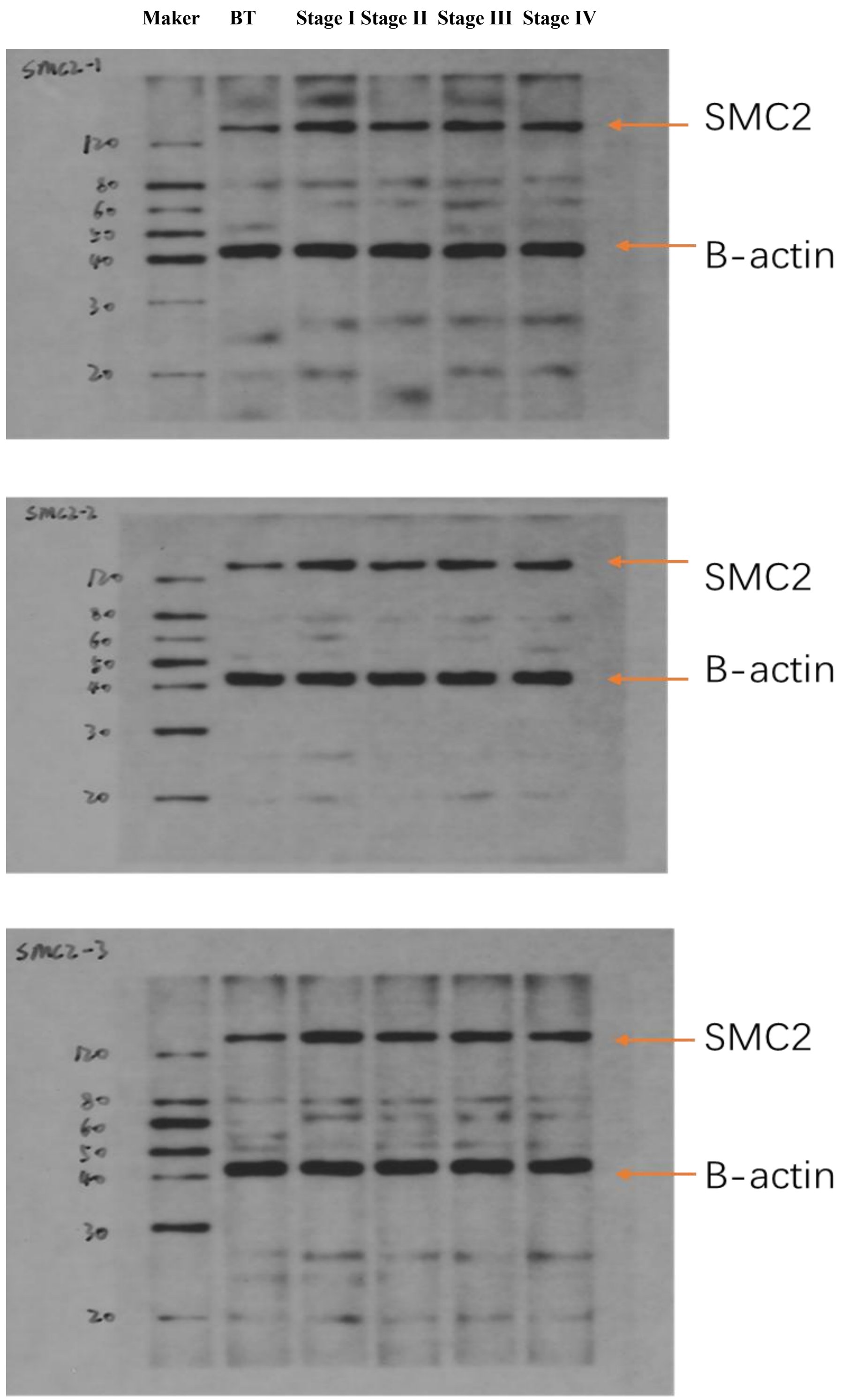

Supplement: Supplementary file 1 [file DataSheet1.pdf]
